# Melanotic Neuroectodermal Tumor of Infancy, a Rapidly Growing Maxillary Alveolar Mass: A Case Report

**DOI:** 10.30476/DENTJODS.2019.44910

**Published:** 2020-03

**Authors:** Saede Atarbashi-Moghadam, Ali Lotfi, Mohammad Moshref, Fazele Atarbashi-Moghadam

**Affiliations:** 1 Dept. of Oral and Maxillofacial Pathology, Dental School of Shahid Beheshti University of Medical Sciences, Tehran, Iran; 2 Dept. of Periodontics, Dental School of Shahid Beheshti University of Medical Sciences, Tehran, Iran

**Keywords:** Neuroectodermal Tumor, Melanotic, Infant, Soft tissue neoplasm, Pigmentation

## Abstract

Melanotic neuroectodermal tumor of infancy is a rare, rapidly growing, painless, pigmented neoplasm with neural crest derivation. It usually occurs during the first year of life and there is a prominent predilection for the maxilla. The purpose of the present report is to describe additional case of melanotic neuroectodermal tumor of infancy of maxilla in a 6-month-old infant male. The treatment included surgical excision with safe margins. No attempt was made for immediate grafting of the surgery site due to high proliferation rate of tissues and self-renewal during infancy. The facial growth was normal and the surgical cleft was tightly closed. Due to the rarity of tumor, essential knowledge on characteristics of this lesion would contribute to a proper diagnosis and benefit treatment planning.

## Introduction

The melanotic neuroectodermal tumor of infancy (MNTI) is a rare pigmented tumor that usually arises during the first year of life. The lesions are more common in the maxilla [ 1 - 4 ]. It has male predilection [ 1 , 4 - 5 ] but some authors mentioned no gender predilection [ 2 - 3 ]. Early diagnosis is necessary owing to rapid disfiguring spread and impact on the adjacent structures [ 1 - 2 ]. The purpose of this paper is to demonstrate a case of MNTI treated without any intervention or grafting. 

## Case Presentation

**Figure1:**
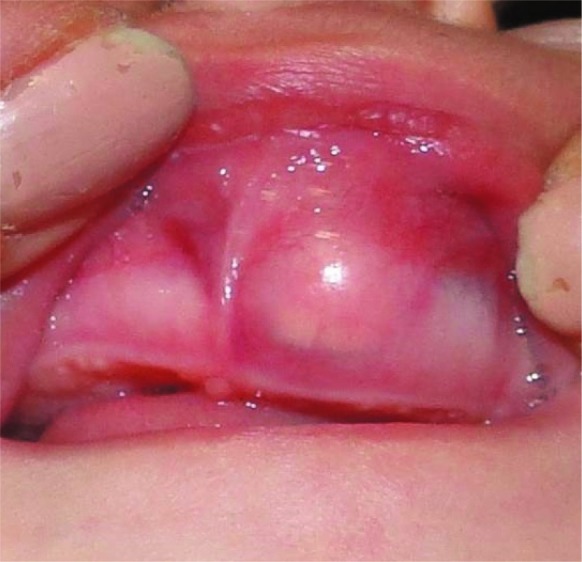
A firm, projecting mass on left maxillary anterior alveolar ridge in a 6-month-old boy

A sixmonthold male was referred to the private oral pathology center (Tehran, Iran) for evaluation of rapidly growing, painless expansion in the left maxilla. His parents noticed the first sign of expansion four months ago without any systemic signs such as fever or malaise. His delivery and development was normal. Upon oral examination, a firm, projecting mass measuring 2.5×2.5×2cm was detected on the left maxillary anterior alveolar ridge extending to the hard palate and displacing the upper lip. It was not tender on palpation. The overlying mucosa was intact and bluish in color (Figure 1). The deciduous teeth were not erupted.

**Figure2:**
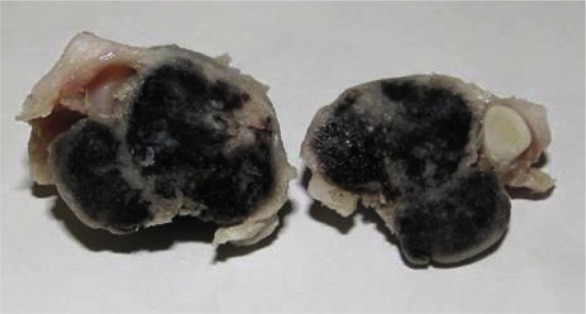
The gross specimen shows a circumscribed mass with black cut surface.

**Figure3:**
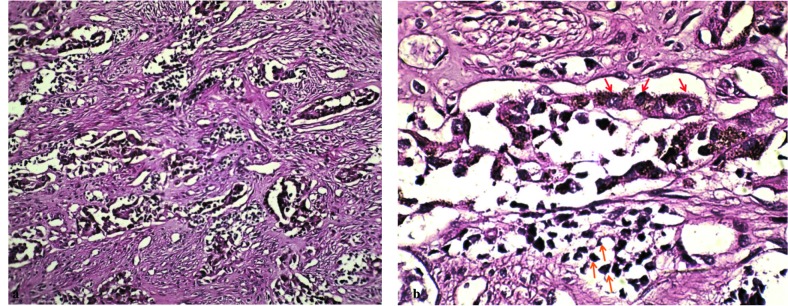
**a:** Section shows a biphasic population of cells that form nests consisting of large epithelioid cells and small rounded neuroblast-like cells ×100. **b:** Large epithelioid cells containing melanin pigmentation (red arrows) and small rounded neuroblast-like cells (orange arrows) ×400

The occlusal radiograph revealed a large radiolucent lesion with ill-defined borders encompassed unerupted tooth buds. With provisional diagnosis of MNTI, the excisional biopsy was performed. Under general anesthesia, a partial maxillectomy with removal of three involved deciduous teeth was done. During surgery, an inner brown-colored aspect of the lesion was observed, which confirmed the initial diagnosis. The lesion was well demarcated from adjacent bone and separated easily. Palatal bone defect was dressed with Surgicell and fixed with absorbable sutures. It is noteworthy that the surgery site was not grafted due to high proliferation rate of tissues and self-renewal during infancy. The gross specimen showed a mass with black cut surface surrounded by a white rim (Figure 2). Microscopic sections showed a biphasic population of cells that form alveolar, nests, and tubules within a collagenous stroma. The alveolar and tubular structures are lined by cuboidal epithelioid cells with vesicular nuclei and granules of brown melanin pigment. The second cell type was small, round cells with hyperchromatic nuclei (Figure 3). The patient presented with excellent clinical features and normal feeding and breathing functions at 6-month follow-up. 

The facial growth has been normal and the palatal cleft was closed, but due to loss of several tooth buds, frequent dental follow-up examinations are essential and this situation may cause facial development problems in future.

## Discussion

MNTI has neural crest origin due to neuroblastic appearance of cells, presence of neurosecretory granules in electron microscopy evaluation, and elevated levels of vanillyl mandelic acid (VMA) in urinary excretion1. However, many cases have been described with normal levels of VMA[2]. These multipotent embryonal cells show mesodermal and ectodermal morphologic features at different stages of their development explaining the biphasic cellular phenotype [ 5 ]. Conventional radiographs usually demonstrate a well-circumscribed, radiolucent, expansile lesion that involves the anterior maxilla with tooth displacement and sometimes with floating in air appearance. Sunburst pattern may be seen but calcification is infrequent. The adjacent bones can be condensed due to reactive sclerosis [ 6 ].

On gross examination, it seems dark blue and there is a suggestion of pseudo-encapsulation because of reactive bone formation [ 4 ]. MNTI is usually firm, lobulated and well circumscribed, compressing rather than infiltrating adjacent components [ 6 ]. Histopathologically, biphasic population of cells that creates nests, tubules, or slit-like alveoli are seen within a moderately vascularized fibrous stroma. 

The alveolar and tubular structures are lined by large cuboidal epithelioid cells, with pale abundant cytoplasm, vesicular nuclei, and granules of brown melanin pigment. The second cell type is neuroblastic or similar to mature lymphocyte and it contains small, round cells with hyperchromatic nuclei and little cytoplasm. These cells are frequently surrounded by the larger pigmented cells [ 7 ].

Microscopic differential diagnosis in this age group contains metastatic neuroblastoma, Ewing sarcoma, rhabdomyosarcoma, primitive neuroectodermal tumor, and lymphoma [ 1 , 5 ].

No histologic findings and biological indicators have been found to highlight the clinical manner [ 4 - 5 , 8 ], albeit, it is alleged that ki-67 and CD99 may predict aggressive behavior [ 5 , 8 ]. Neuron-specific enolase (NSE) expression could potentially lead to poor prognosis in neuroblastoma, but such a finding has not been seen in MNTI [ 8 ]. Moreover, there is a slight alteration in the microscopic features of the primary and the recurrent lesion and in metastasis; the smaller round cells is predominant [ 1 , 4 , 8 ]. The epithelioid cells are positive for cytokeratin, HMB-45, and NSE and the smaller cells usually are positive for NSE and CD56, and sometimes synaptophysin [ 1 , 8 ]. Melanocytes are expressed S-100 protein but MNTI is negative for this marker, which might indicate an unusual phenotypic profile. The larger epithelioid cells that express MDM-2, cyclin D1, and cyclin A, eventually propose that these cells are the proliferative component of MNTI [ 8 ].

Generally, the prognosis of MNTI is excellent [ 2 ]. Surgical excision, without an incisional biopsy, can be achieved based on clinical and radiological outcomes [ 6 ]. Wide surgical margins may not be necessary as tumor islands may degenerate spontaneously after inadequate excision whereas others recommended tumor resection with wide surgical excision of at least 5mm [ 4 , 6 , 9 ]. Peripheral tumor cells rely on a group of motivating cells in the center of the lesion. In the absence of central stimulating cells, peripheral cells are doomed to death [ 7 ]. Recurrence has been reported in about 20% of cases that occurred within 4 weeks after the operation [ 1 , 3 ]. 

The recurrent MNTI appears to grow more destructively, to have a propensity for indistinct borders, and it might demonstrate osteoid formation [ 9 ]. Adjuvant chemotherapy has also been suggested for recurrent MNTI but adverse effects of chemotherapy in infants must be taken into great consideration [ 3 ]. Radiotherapy is ineffective. Malignancies are typically only established by the existence of metastasis [ 4 ] and a few malignant tumors have been reported that especially occurred in brain and skull [ 1 ]. Rachidi et al. [ 5 ] claimed that age of the patient at the time of diagnosis is an important prognostic factor. Higher recurrence rates were seen in patients diagnosed during the first two month of life. Monthly follow up is mentioned during the first postoperative year [ 9 ].

All appropriate patient consent forms were obtained from his parents.

## Conclusion

In our case, MNTI indicated a benign course, and the surgical cleft was closed without any grafting that indicate high proliferation rate in neonates. 
